# The Effect of Closed‐Loop Auditory Stimulation on Memory Consolidation and Sleep Physiology in an Ecological Setting

**DOI:** 10.1111/jsr.70247

**Published:** 2025-11-12

**Authors:** Angie Baldassarri, Damiana Bergamo, Federico Salfi, Domenico Corigliano, Michele Ferrara, Aurora D'Atri, Nicola Cellini

**Affiliations:** ^1^ Department of General Psychology University of Padua Padua Italy; ^2^ Department of Biotechnological and Applied Clinical Sciences University of L’Aquila L'Aquila Italy; ^3^ Padova Neuroscience Center University of Padua Padua Italy; ^4^ Human Inspired Technologies Research Center University of Padua Padua Italy

**Keywords:** closed‐loop auditory stimulation, declarative memory consolidation, ecological setting, language learning, slow wave sleep

## Abstract

Slow wave sleep plays a crucial role in overnight memory consolidation, with slow oscillations serving as a critical mechanism. Recent studies have identified closed‐loop auditory stimulation as an effective method to enhance slow oscillatory activity during slow wave sleep, thereby facilitating memory consolidation. However, few studies have applied this technique in ecological settings, with inconsistent findings. The present study investigated the short‐ and long‐term effects of closed‐loop auditory stimulation on declarative memory performance and vigilance. Additionally, we examined potential sleep microstructural changes. A between‐subjects design was employed on 34 participants who were divided into a Control group and a Stimulation group, the latter receiving one night of closed‐loop auditory stimulation in a home environment. While stimulation successfully enhanced slow oscillation amplitude, no behavioural effects on memory performance or vigilance were observed. However, these findings should be interpreted with caution, as our limited sample size may have been insufficient to detect a potential effect of CLAS on memory.

## 1 Introduction

It is now well‐established that sleep plays a critical role in memory consolidation, with research showing that a period of sleep benefits memory more than an equivalent period spent awake (Diekelmann and Born 2010; Rasch and Born 2013). In particular, slow wave sleep (SWS) has been highlighted for its importance in this process. During SWS, memory traces are reactivated and reorganised within cortical networks, where they integrate with pre‐existing information and become independent from the hippocampus (Born and Wilhelm 2012). This process relies on the temporal coordination of specific oscillatory events, such as slow oscillations (SOs), sleep spindles, and ripples, that facilitate the communication between neocortical and hippocampal areas.

Recently, several studies have attempted to manipulate sleep oscillations, particularly SOs, to better understand their impact on memory (see Cellini and Mednick 2019). Among studies employing auditory stimulation (e.g., Ngo et al. 2013, 2015; Ong et al. 2016, 2018; Papalambros et al. 2017; Shimizu et al. 2018) closed‐loop auditory stimulation (CLAS) has emerged as a promising technique, involving the application of a brief auditory stimulus, often a 50 ms pink noise, time‐locked to a particular phase of the SO (see Bellesi et al. 2014; Cellini and Capuozzo 2018). Previous research suggests that stimulation during the up‐state of the SO produces physiological effects, such as enhancing SO amplitude and eliciting SO trains, as well as memory improvements (see Esfahani et al. 2023; Navarrete et al. 2020). However, when considering closed‐loop stimulation approaches, the evidence appears mixed: while some studies report beneficial effects, others have failed to detect any significant impact of CLAS on memory performance in young adults, either during a nap or overnight sleep (Henin et al. 2019; Schneider et al. 2020).

While laboratory studies have mainly shown beneficial effects of CLAS on both sleep microstructure and cognitive functions, it remains unclear whether these results can be replicated in real‐world settings. Only a few studies have applied CLAS using wearable electroencephalography (EEG) devices in home environments, such as the Dreem Headband (Debellemaniere et al. 2018) and the MHSL‐Sleepband V2 (Ferster et al. 2019), demonstrating stimulation accuracy comparable to that achieved in laboratory studies (Debellemaniere et al. 2018; Ferster et al. 2019; Garcia‐Molina et al. 2018). These studies reported changes in sleep microstructure induced by the stimulation, primarily characterised by an increase in slow‐wave activity (0.5–4 Hz) (Lustenberger et al. 2022). However, effects on memory performance were rarely assessed, with Diep et al. (2021) being the only study to investigate this aspect without finding any effect.

On the other hand, the enhancement of SWS oscillatory events is thought to improve daytime sleep‐related performance, with psychomotor vigilance being one of the most sensitive to detect sleep‐related performance impairments (Durmer and Dinges 2005). However, findings on psychomotor vigilance appear to be more controversial, with effects of CLAS reported only in chronically sleep‐restricted participants (Diep et al. 2021).

In light of this literature, we conducted a between‐subjects study aimed to determine whether a single night of auditory stimulation in an ecological setting, applied using a wearable device, could benefit declarative memory consolidation and vigilance the following morning. Additionally, we aimed to observe whether effects on memory performance would persist 2 days after the stimulation. Besides its effect on cognitive functions, we expected that CLAS would lead to changes in sleep microstructure, specifically by inducing a second SO following the stimulation, unlike the non‐stimulated SOs.

## 2 Materials and Methods

### 2.1 Participants

Thirty‐eight participants between the ages of 18 and 35 took part in the experiment. The sample size was determined based on feasibility constraints, as the EEG device used in the study was no longer available, which prevented the recruitment of additional participants.

All participants completed an online questionnaire via Google Forms to provide general demographic information, along with details on psychological symptoms and sleep habits that could potentially influence their memory performance. The only inclusion criterion was that participants had to be native Italian speakers. Besides this, we did not apply other inclusion/exclusion criteria, as the study aimed to assess the effects of CLAS in the general population. They were instructed to maintain stable sleep patterns throughout the experiment. To monitor compliance, participants completed a daily sleep diary each morning, starting from the second day of the experiment.

Four participants were excluded from the analyses due to issues with the sleep recording device or poor signal quality. Therefore, the final sample consisted of 34 participants (Mean age = 25.09, SD = 3.96 years, 15 males).

The study protocol was approved by the Ethics Committee for Psychological Research, Area 17 of the University of Padua, in accordance with the principles of the Helsinki Declaration.

### 2.2 Procedure

Before starting the experiment, each participant digitally signed an informed consent form. Two days before the experiment, they completed a series of screening online questionnaires via Google Forms. Specifically, the administered questionnaires included: the Epworth Sleepiness Scale (ESS; Johns 1991; Vignatelli et al. 2003) to assess excessive sleepiness, the Pittsburgh Sleep Quality Index (PSQI; Buysse et al. 1989; Curcio et al. 2013) to assess the presence of sleep disturbances, the Insomnia Severity Index (ISI; Bastien 2001; Castronovo et al. 2016) to assess insomnia symptomatology, the Morningness–Eveningness Questionnaire reduced version (MEQ‐r; Adan and Almirall 1991; Natale et al. 2006) to assess circadian preferences, the Depression, Anxiety and Stress Scale‐21 Items (DASS‐21; Henry and Crawford 2005; Bottesi et al. 2015) to assess distress symptomatology, and the Multifactorial Memory Questionnaire (MMQ; Troyer and Rich 2002; Raimo et al. 2016) to assess metamemory abilities.

After completing the questionnaires, the experimenter met the participants to deliver the Dreem Headband 2 (DH2; Rythm SAS, Paris, France), a wireless electroencephalography (EEG) device, where they were instructed on the proper use of the device.

The experiment lasted 5 days and was conducted entirely at the participants' homes. For task administration, participants connected with the experimenter via Zoom, where they received the link to the Pavlovia online platform (https://pavlovia.org/), through which the tasks were delivered. The experimenter remained present throughout to ensure compliance and clarify instructions if needed.

The study employed a between‐subjects design with two groups: the Stimulation group (17 participants) and the Control group (17 participants), assigned to the group based on recruitment order. In the Stimulation group, participants experienced a night of CLAS, while the Control group underwent identical procedures, except that no acoustic stimulation was administered.

**FIGURE 1:**
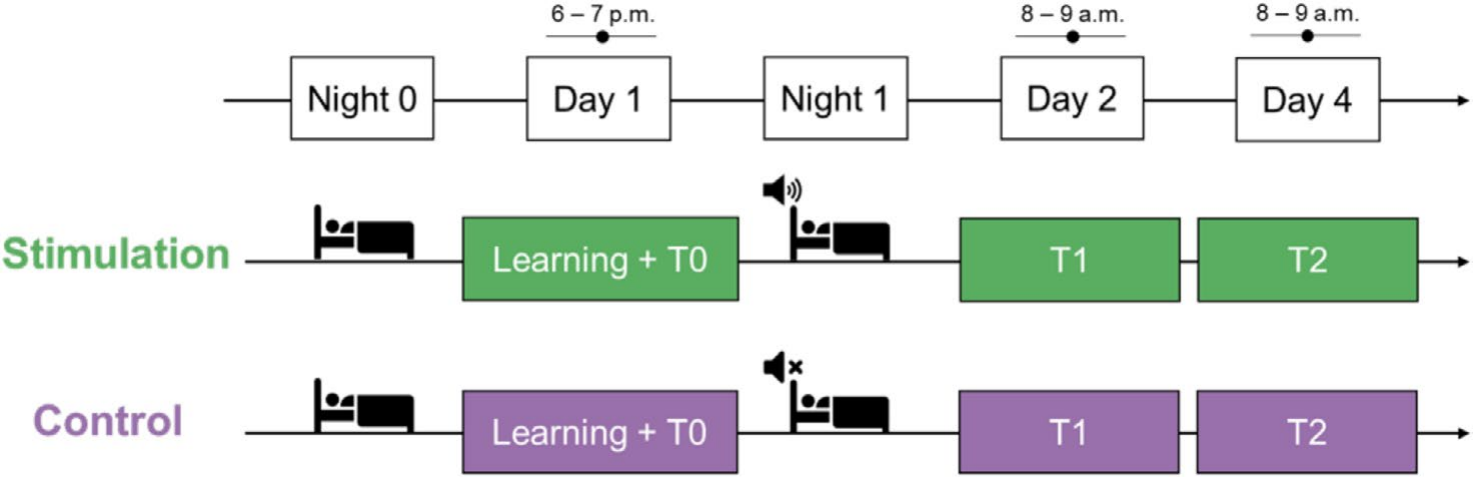
Representation of the experimental procedure. On the adaptation night (Night 0), participants slept with the ambulatory device for adaptation. On the first day, around 6–7 p.m., they underwent the Encoding, Training and Testing (T0) phases. That night, they slept again with the ambulatory device; the Stimulation group received CLAS, while the Control group only had their sleep recorded. On the second day, around 8–9 a.m., they were tested (T1). On the fourth day, they were tested again (T2).

The experimental procedure is illustrated in Figure 1.

On the initial night, participants in both groups slept with the DH2. This night was designated as an adaptation period to allow participants to acclimate to sleeping with the device, without delivering any acoustic stimulation. The following evening (6–7 p.m.), participants completed two different tasks: a psychomotor vigilance task and a word‐paired association task. The word‐pair association task included a learning phase followed by an immediate testing phase (T0). This session lasted approximately 45 min. That night, participants slept with the DH2, with only the Stimulation group receiving acoustic stimulation. The next morning (8–9 a.m.), participants completed both the psychomotor vigilance task and the memory test (T1). This session lasted about 15 min. On the final day (T2; 8–9 a.m.), participants underwent the same tests as they did during T1. Before each task session, the Karolinska Sleepiness Scale (KSS; Åkerstedt and Gillberg 1990) was administered to assess individuals' subjective sleepiness at the time of task administration.

### 2.3 Experimental Tasks

Participants completed a 5‐min Psychomotor Vigilance Task (PVT; Loh et al. 2004) to assess their current level of psychomotor vigilance, requiring them to press the space bar as soon as numbers appeared on the screen. The maximum number of trials was set to 100, although it varied across participants and ended after 5 min. The inter‐stimulus interval (ISI) ranged from 2 to 10 s.

The memory task used in this study is the Word–Pseudoword Association Learning task (WPAL), a word‐paired association task previously used in Salfi et al. (2025). In this task, participants learned the Italian ‘translation’ of 40 pseudowords. The WPAL has two versions (set A and set B) to which participants were assigned in a counterbalanced order.

**FIGURE 2:**
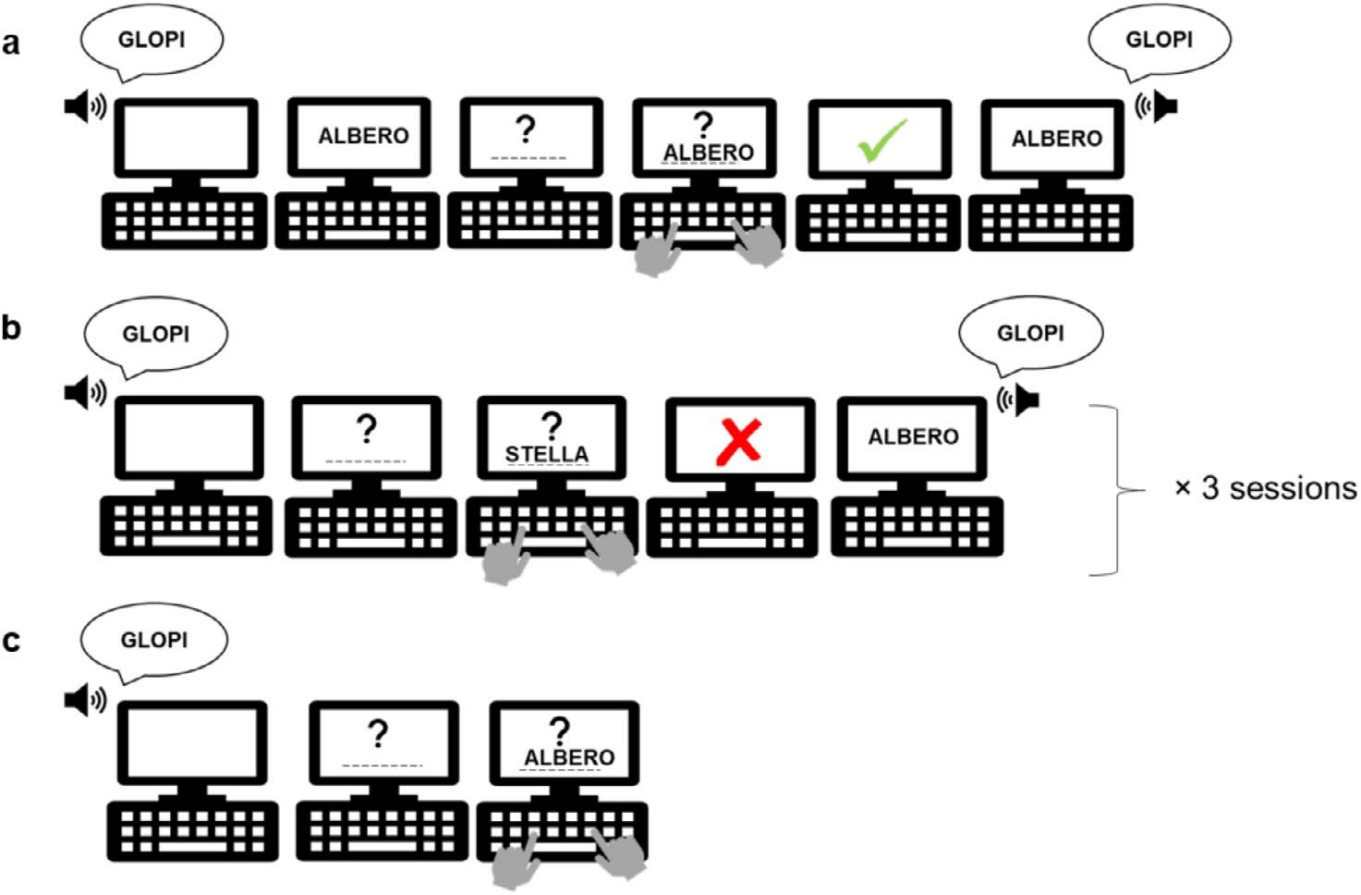
Task representation. (a) Representation of the Encoding phase of the WPAL. Participants heard 40 pseudowords and saw their Italian translations. Then, they were asked to write down the Italian translation they had just seen. Afterward, they received feedback, and the word–pseudoword pairs were presented again simultaneously. (b) Representation of the Training phase of the WPAL. Participants heard 40 pseudowords and were required to recall their Italian translations. They received feedback, and the word–pseudoword pairs appeared again simultaneously. This Training phase was repeated three times. (c) Representation of the Testing phase of the WPAL. Participants heard 40 pseudowords and had to recall their Italian translations. No feedback was provided.

The WPAL consisted of three phases. In the Encoding phase (Figure 2a), participants heard 40 pseudowords followed by their written Italian translation, wrote down the translation, received feedback (correct or incorrect), and saw the pair again. Next, participants entered a Training phase (Figure 2b) where they listened to the 40 pseudowords and had to recall their Italian translations. Feedback and re‐presentation of the word–pseudoword pairs accompanied each recall attempt. This training was repeated three times (Training 1, Training 2, Training 3) with a 1‐min break between each repetition. The final phase was the Test phase (T0; Figure 2c), similar to the training phase but without any feedback. On subsequent days, only the test phase was administered. For each phase, participants were given 10 s to respond. Pair presentation order was randomised in all phases. Both the PVT and WPAL tasks were created in Psychopy 2 (Peirce et al. 2019) and presented online via Pavlovia. For the PVT, the mean reaction time (ms) was assessed. For the WPAL, every participant's answer was categorised as 1 if correct or 0 if incorrect. Misspelled words were corrected and considered accurate.

### 2.4 Sleep Recording and Stimulation Procedure

Participants slept for two nights at home wearing the DH2, a wireless EEG device validated for reliable physiological signal acquisition and sleep staging, with scoring accuracy comparable to five sleep experts (Arnal et al. 2020).

The DH2 records five different types of physiological signals: brain activity (via dry EEG electrodes), movement, body position, respiratory rate, and heart rate. Using these data, the DH2 can automatically stage sleep through a validated automatic scoring system, providing sleep parameters that are accessible through a dedicated cloud service. The DH2 includes five dry electrodes (O1, O2, Fp1, F7, F8) yielding seven bipolar EEG derivations (Fp1–O1, Fp1–O2, Fp1–F7, F8–F7, F7–O1, F8–O2, Fp1–F8). The signal is acquired at a sampling rate of 250 Hz.

The DH2 also features an audio system in the frontal band on the forehead that delivers sounds via bone conduction, enabling the application of CLAS during the N3 sleep stage (see Debellemaniere et al. 2018). To detect a SO to stimulate, the DH2 employs a phase fitting algorithm inspired by Cox et al. (2014) on Fp1–O1 and Fp1–O2 derivations, which fits several sinusoids to the filtered EEG signal (0.4–4 Hz) to estimate in real time the current phase of the SO. The stimulation consists of a 100 ms pink noise stimulus, with the volume randomly set at either 45 or 55 dB, varying between subjects. Five of the 17 participants (29%) received pink noise at 55 dB, while the remaining received 45 dB. If another SO occurs within 2 s, a consecutive pink noise stimulus is delivered. The device starts to send stimulations only after 15 min of stable N3 sleep, with a minimum pause of 9 s between stimulation trains. If movements or alpha rhythms are detected within 6 s following a stimulation, the system imposes a 30‐s pause before attempting further stimulations. The number of stimuli received varies across participants, depending on the number of detected SOs. During stimulation nights, approximately 22% of triggers are sham (see Table S4), meaning the stimulation time point is marked, but no sound is sent.

Activation or deactivation of stimulation was controlled through the DH2 mobile application. For this reason, participants were not blind to the condition, as they were required to select the stimulation mode themselves.

Participants in the stimulation group (*N* = 17) received a mean of 332.6 (SD = 137.7) stimulations and a mean of 95.0 (SD = 38.4) sham triggers (see Table S4).

### 2.5 Preprocessing and Analyses of Sleep EEG Data

To assess the impact of stimulation on EEG activity, event‐related potentials (ERPs) locked to each trigger sent during the stimulation night were computed. Triggers were categorised into six types: three related to the Stimulation condition (Stim 1, Stim 2, Unique Stim) and three related to the Sham condition (Sham 1, Sham 2, Unique Sham). Stim 1 and Sham 1 refer to the first trigger in a consecutive pair, while Stim 2 and Sham 2 refer to the second trigger in the pair. The Unique Stim and Unique Sham occurred in isolation. For the ERPs, Stim 1 and Unique Stim, as well as Sham 1 and Unique Sham, were combined as First‐type triggers to enable the analysis of first‐trigger responses.

DH2 raw signal (0.4–35 Hz) was low‐pass filtered at 18 Hz and segmented into 12 s epochs centred around each trigger. Data segments with clear artefacts (e.g., signal loss, sweating‐related drifts or electrode detachment) were manually rejected based on visual inspection of the F7–O1 channel. Channel F7–O1 was chosen based on preliminary visual inspection, as it appeared cleanest in most subjects among occipital‐referenced channels, maximising the number of usable epochs. ERPs were computed using a baseline correction from −4 to −3 s.

Then, for each subject, the negative peak amplitudes around the First‐type Stim triggers were computed for both negative peaks in the ERP, one occurring before and one after the trigger. The first minimum negative peak was identified before the trigger, while the second was detected within 200 timepoints after the trigger. For each subject, the ratio between the negative peak after stimulation and the one before was calculated, reflecting the amplitude difference between stimulated and spontaneous SOs. This difference (Δnegativepeak) was computed as $\left(\frac{\text{NegativePeak}\_\text{afterstim}}{\text{NegativePeak}\_\text{beforestim}}\right)\times 100$. In addition, Δpeaktopeak was computed as the percentage change between the up‐to‐down peak‐to‐peak amplitudes after the trigger in the First‐type Stim and First‐type Sham conditions: $\left(\frac{\text{PeaktoPeak}\_\text{Stim}}{\text{PeaktoPeak}\_\text{Sham}}\right)\times 100$.

We did not conduct individual spindle or SOs detection because we judged the signal quality insufficient for accurate single‐event detection, mainly due to sweating artefacts and signal loss in the occipital electrodes, which often did not adhere completely to the head.

EEG data preprocessing and analyses were performed using the EEGLAB toolbox (version 2024.2; Delorme and Makeig 2004) along with custom MATLAB scripts (R2022a; https://www.mathworks.com).

### 2.6 Statistical Analyses

Sample demographics, questionnaire scores, and sleep parameters from the experimental night were reported with mean and standard deviation for both groups and compared using independent samples *t*‐tests (*t*), Mann–Whitney tests (*U*), or chi‐square tests (*χ* ^2^). MEQ‐r scores were categorised as Evening‐type (scores 4–10), Intermediate‐type (scores 11–18), and Morning‐type (scores 19–25), according to their Italian validation (Natale et al. 2006).

Memory performance was assessed based on the binary outcome (correct/incorrect) of word–pseudoword pairs recall. Changes over time were expressed as percentage variation in accuracy between sessions (e.g., ΔT1T0 is computed as $\frac{T1}{T0}\times 100$).

PVT trials with reaction times < 100 ms or > 500 ms were excluded. To assess possible changes in psychomotor vigilance, a mixed‐effects generalised linear model (GLM) with Gamma distribution was conducted on single‐trial PVT reaction times, with Group (Control, Stimulation) and Session (T0, T1) as fixed effects, and participants as a random effect. The analysis of PVT was conducted excluding the T2 session, as we did not expect any long‐term CLAS effect on vigilance.

The training trajectory was examined through a mixed‐effects logistic regression model with a binomial distribution. The dependent variable was the participants' responses, while the fixed effects included Session (Training 1, Training 2, Training 3, T0) and Group (Control, Stimulation). Participants and Italian words were included as random effects.

The same model was applied to assess changes in memory performance during testing phases, with Group (Control, Stimulation) and Session (T0, T1, T2) as fixed effects, and with participants and Italian words as random effects.

To account for CLAS effects on memory without the influence of possible differences in memory performance between groups at T0, Mann–Whitney tests were conducted to compare memory performance changes over time (e.g., ΔT1T0) between the two groups.

To ensure that CLAS actually induced physiological changes, within‐subject comparisons of ERPs (Stim and Sham conditions) on channel F7–O1 using a smaller time window (−1.5 to 1.5 s) were performed. A permutation test with 5000 randomizations was conducted, and cluster‐based correction was applied to control for multiple comparisons. Paired *t*‐tests were computed for each time point (4 ms), and contiguous time points with *p* < 0.05 were grouped into clusters. Condition labels were randomly permuted within participants to build the null distribution of cluster *t*‐sums. Clusters were considered significant if their *t*‐sum exceeded the 97.5th percentile or fell below the 2.5th percentile of the null distribution. For each significant cluster (*p* < 0.05, two‐tailed), the sum of the *t* values (*t*‐sum) and the cluster‐level *p*‐value were reported.

To verify the accuracy of the timing of stimulations, we conducted a phase analysis of the stimulation triggers in the stimulation group. This analysis is described in the Supporting Information.

To rule out CLAS‐related arousal, the micro‐arousal index (micro‐awakenings per hour) was compared between adaptation and experimental nights in the Stimulation group using the Wilcoxon signed‐rank test. Spearman correlations examined relationships between ΔT1T0 and both Δnegativepeak and Δpeaktopeak in the Stimulation group. Kendall correlations were conducted to explore possible relationships between KSS outcomes and mean performance on the PVT for each session, as well as between KSS and memory percentage accuracy at T0. These analyses were performed to rule out the possibility that differences in PVT performance and memory accuracy were due to variations in participants' sleepiness at the time of testing.

Post hoc tests were corrected using Holm's correction method.

Effect sizes were reported as Cohen's *d* (*t*‐tests), Wilcoxon's *r* (Mann–Whitney, Wilcoxon test), Cramer's *V* (chi‐square tests), odds ratios (logistic regression), or exponentiated coefficients (*b*; Gamma GLM). Analyses were conducted both with and without influential observations; however, as the results did not differ and there was no compelling reason for their removal, they were retained in the final analyses.

All statistical analyses were conducted using RStudio (R Core Team 2023) with *p* < 0.05 as the significance threshold.

## 3 Results

### 3.1 Demographics

Table 1 shows the demographic characteristics and the results of the questionnaires, separately for the two groups. A comparison between the Control and the Stimulation group is reported. No significant differences were found between groups. P values reported below are not adjusted for multiple comparisons (all adjusted *p*'s = 0.999).

**TABLE 1:** Demographic and trait characteristics of the two groups.

	Control (*n* = 17)	Stimulation (*N* = 17)	Test	*p*	Effect size
Age (years)	25.2 ± 3.9	24.9 **±** 4.1	*t* = 0.21	0.832	*d* = 0.07
Gender (F/M)	10/7	9/8	*χ* ^2^ < 0.001	0.999	*V* = 0
Dass‐21 anxiety	3.1 ± 3.6	3.6 **±** 4.3	*U* = 127.50	0.562	*r* = 0.10
DASS‐21 depression	4.0 ± 4.8	4.9 ± 4.5	*U* = 116.00	0.331	*r* = 0.20
DASS‐21 stress	5.7 ± 4.8	7.2 ± 5.7	*U* = 126.50	0.544	*r* = 0.12
PSQI	6.2 ± 2.4	7.1 ± 2.0	*t* = −1.17	0.248	*d* = −0.40
ISI	12.6 ± 2.3	13.0 ± 3.2	*U* = 134.50	0.738	*r* = 0.06
MEQ‐r (I/M/E)^a^	10/4/3	9/2/6	—	0.419^b^	*V* = 0.22
ESS	4.8 ± 2.9	5.1 ± 2.4	*t* = −0.26	0.795	*d* = −0.09
MMQ satisfaction	48.5 ± 8.6	48.9 ± 10.8	*U* = 134.00	0.730	*r* = 0.06
MMQ ability	54.4 ± 12.5	54.8 ± 10.9	*t* = −0.10	0.919	*d* = −0.03
MMQ strategy	36.3 ± 12.1	32.3 ± 11.6	*t* = 0.98	0.332	*d* = 0.34

*Note:* Mean ± standard deviation of demographic and trait variables.

Abbreviations: DASS‐21: Depression, Anxiety and Stress Scale‐21 Items; ESS: Epworth Sleepiness Scale; ISI: Insomnia Severity Index; MEQ‐r: Morningness–Eveningness Questionnaire reduced version; MMQ: Multifactorial Memory Questionnaire; PSQI: Pittsburgh Sleep Quality Index.

^a^ Intermediate‐type/Morning‐type/Evening‐type.

^b^ Fisher's exact test.

*P*'s shown are not adjusted for multiple comparisons.

### 3.2 Psychomotor Vigilance

The model conducted on PVT reaction times did not reveal any difference between groups at T0. A difference between T0 and T1 was present in the Control group (see Table 2), with higher reaction times at T1 (*b* = 1.02, SE = 0.01, *z* = 3.11, *p* = 0.002). No significant Group × Session interaction was observed, although reaction times increased in the Control group, whereas they remained stable in the Stimulation group (for a graphical representation, see Figure S1). Full model results can be found in Table S1.

**TABLE 2:** Mean ± standard deviation of mean PVT reaction time (ms) across the two sessions in the two groups.

	T0	T1
Stimulation (*N* = 17)	320.41 ± 53.08	320.85 ± 67.04
Control (*N* = 17)	316.76 ± 45.58	324.54 ± 47.36

### 3.3 Memory Task

Results of analyses conducted on training trajectory can be found in Table S2.

**FIGURE 3:**
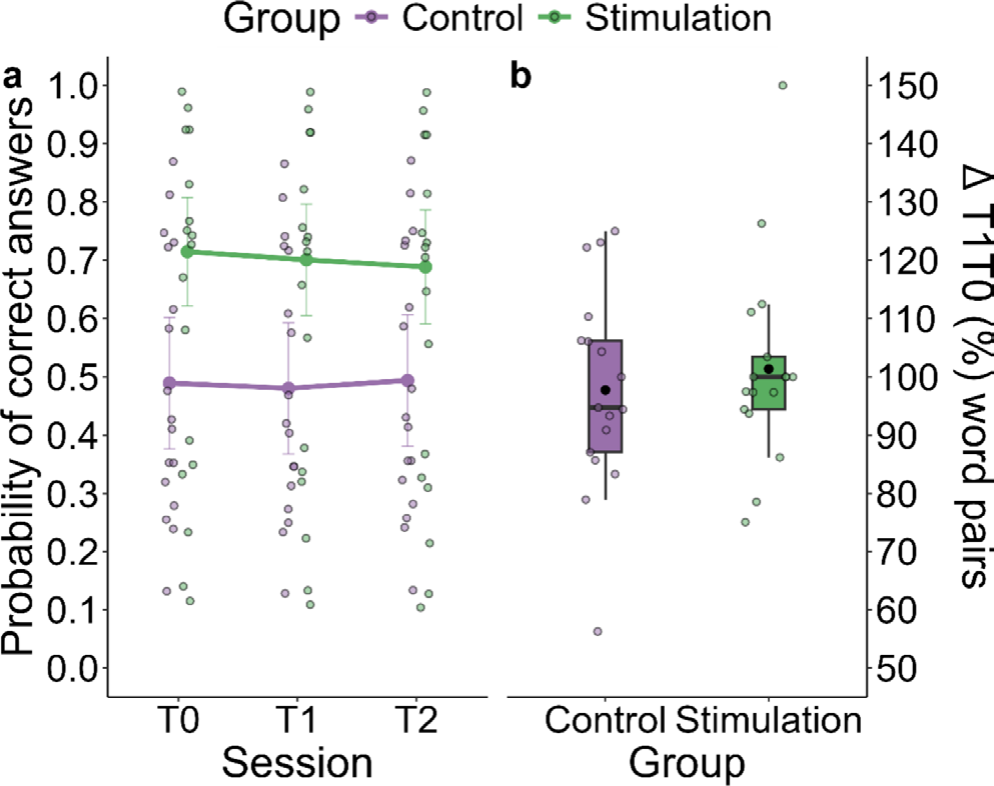
Memory task performance. (a) Mean probability of correct answers for word–pseudoword pairs in the two groups (Control, Stimulation) during the testing phases. Larger dots represent the mean probability across subjects. Smaller dots represent individual participants' observed data. Bars represent standard errors. (b) Changes in word–pseudoword pairs accuracy between T0 and T1 (ΔT1T0) in the two groups. Each dot represents an individual participant's observed data. The black dot represents the mean value. The box indicates the interquartile range (IQR), and the horizontal line is the median. Bars outside the box represent 1.5 × IQR.

The analysis of word–pseudoword pairs during the testing phases showed no significant differences between groups at T0 (*b* = 2.61, SE = 1.61, *z* = 1.56, *p* = 0.119). Additionally, there were no differences between sessions (T1–T0; *b* = 0.96, SE = 0.13, *z* = −0.27, *p* = 0.787, T2–T0; *b* = 1.02, SE = 0.14, *z* = 0.14, *p* = 0.893), nor any significant interaction between Group and Session (see Figure 3a). Full model results can be found in Table S3.

The Mann–Whitney test comparing ΔT1T0 accuracy for word–pseudoword pairs between the Stimulation and Control groups did not reveal any significant difference (*U* = 157.5, *p* = 0.666, *r* = 0.07; Figure 3b).

### 3.4 Sleep Macrostructure

Among the 34 subjects included in the analyses, data from two nights (one adaptation night and one experimental night) from two different participants in the Control group were discarded due to incomplete EEG recordings. Table 3 presents the main sleep parameters from the Experimental night for each group, described by mean and standard deviation. Comparisons between groups did not reveal any significant difference between the two groups. *P* values in the table are not adjusted (all adjusted *p*'s = 0.999).

**TABLE 3:** Mean and standard deviation of sleep parameters during the experimental night and comparison between the two groups.

	Control (*N* = 16)	Stimulation (*N* = 17)	Test	*p*	Effect size
TIB (min)	447.1 ± 81.6	456.2 ± 46.7	*t* = −0.39	0.692	*d* = −0.14
TST (min)	420.0 ± 75.4	419.9 ± 49.7	*t* = 0.002	0.997	*d* < 0.01
SOL (min)	11.0 ± 5.2	12.8 ± 8.2	*U* = 122.5	0.639	*r* = 0.08
WASO (min)	14.8 ± 8.5	23.1 ± 17.9	*U* = 105.5	0.279	*r* = 0.19
Awakenings N3 (N)	3.06 ± 1.5	2.47 ± 1.2	*t* = −1.24	0.223	*d* = −0.43
SE (%)	94.0 ± 3.2	91.9 ± 3.9	*U* = 178.0	0.136	*r* = 0.26
N1 (min)	24.9 ± 8.0	23.9 ± 9.1	*t* = 0.31	0.752	*d* = 0.11
N2 (min)	197.5 ± 52.8	185.6 ± 42.4	*t* = 0.71	0.482	*d* = 0.24
N3 (min)	94.8 ± 28.1	93.6 ± 27.3	*t* = 0.13	0.900	*d* = 0.04
REM (min)	104.6 ± 33.8	116.8 ± 31.5	*t* = −1.07	0.291	*d* = −0.37
NREM (min)	315.4 ± 52.3	303.1 ± 31.6	*U* = 169.0	0.121	*r* = 0.27
N1 (%)	5.9 ± 1.3	5.8 ± 2.5	*U* = 152.0	0.369	*r* = 0.16
N2 (%)	46.7 ± 8.0	43.9 ± 6.6	*t* = 1.08	0.289	*d* = 0.38
N3 (%)	23.3 ± 8.0	22.8 ± 7.8	*t* = 0.19	0.845	*d* = 0.07
REM (%)	24.5 ± 4.9	27.5 ± 5.4	*t* = −1.68	0.102	*d* = −0.58
NREM (%)	75.8 ± 4.9	72.5 ± 5.4	*t* = 1.80	0.081	*d* = 0.63

*Note:* Awakenings N3 are the number of transitions from N3 sleep to wakefulness.

Abbreviations: SE: sleep efficiency; SOL: sleep‐onset latency; TIB: time in bed; TST: total sleep time; WASO: wake after sleep onset.

*P*'s shown are not adjusted for multiple comparisons.

### 3.5 ERP Analysis

**FIGURE 4:**
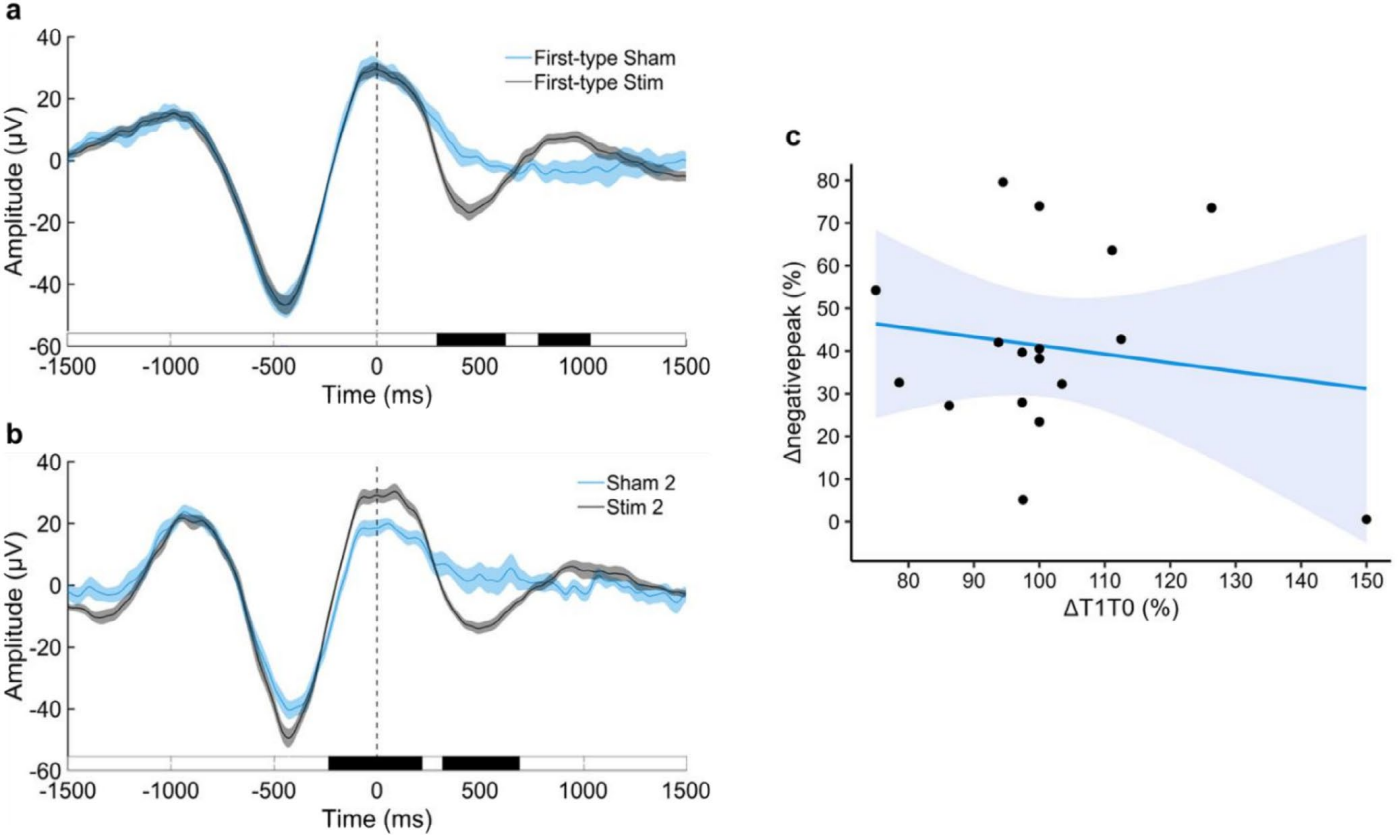
Effects of auditory stimulation on SOs. The black bar indicates the timepoints with a significant difference (*p* < 0.05) between Stim (black line) and Sham (blue line) conditions, based on analysis performed on 17 subjects. Each bin is 4 ms long. Standard error is indicated by the shaded areas. (a) Grand‐average of the EEG signal around the First‐type trigger (i.e., Stim 1 + Unique Stim, Sham 1 + Unique Sham). Time 0 = stimulation onset. (b) Grand‐average of the EEG signal around the second trigger (i.e., Stim 2, Sham 2). Time 0 = stimulation onset. (c) Scatter plot showing the relationship between Δnegativepeak in the First‐type Stim trigger and ΔT1T0 percentage accuracy in the Stimulation group.

The comparison of the averaged EEG amplitude time‐locked to the First‐type trigger (i.e., Stim 1 + Unique Stim, Sham 1 + Unique Sham) on the F7–O1 channel was conducted on a total of 5158 (*M*_per subject_ = 303.4, SD = 127.1) and 1484 (*M*_per subject_ = 87.3, SD = 36.6) epochs, respectively. Compared to the First‐type Sham condition, the First‐type Stim condition shows a second SO induced by the stimulation, characterised by a down‐state between 292 and 620 ms after the stimulation (*t*‐sum = 348.07, *p* < 0.001) and a second up‐state between 784 and 1032 ms after the trigger (*t*‐sum = −185.69, *p* = 0.019) (see Figure 4a). Regarding the averaged EEG amplitude time‐locked to the second trigger (i.e., Stim 2, Sham 2) on the F7‐O1 channel, the comparison was conducted on a total of 4466 (*M*_per subject_ = 262.7, SD = 116.9) and 1243 (*M*_per subject_ = 73.1, SD = 33.1) epochs, respectively. From the ERP on the second trigger, we can observe a difference between Stim and Sham conditions before the stimulation, with the Stim 2 condition showing a greater amplitude in the up‐state between 236 ms before and 212 ms after the trigger (*t*‐sum = −409.18, *p* < 0.001). Additionally, Stim 2 induced a down‐state between 316 and 684 ms after the stimulation trigger (*t*‐sum = 366.38, *p* < 0.001) (see Figure 4b).

Of note, the computation of the difference in up‐to‐down peak‐to‐peak amplitude (Δpeak‐to‐peak) between the First‐type Stim and First‐type Sham conditions revealed that only 11 out of 17 participants exhibited a greater SO negative peak in the Stim condition compared to the Sham condition (see Table S5). Nevertheless, the one‐sample *t*‐test comparing Δpeak‐to‐peak to 100 confirmed that, on average, the peak‐to‐peak amplitude was higher than 100%, indicating a generally successful effect of the stimulation (*t*_16_ = 2.45, *p* = 0.026, *d* = 0.59).

### 3.6 Memory Performance and Characteristics of Evoked SOs

An exploratory correlation analysis was performed to examine the relationship between ΔT1T0 percentage memory change and First‐type Stim Δnegativepeak within the Stimulation group. No significant correlation was found (*r*_s15_ = −0.006, *p* = 0.981), indicating no relationship between the change in memory performance between T1 and T0 and the difference between the negative peak amplitude of stimulated SOs and spontaneous SOs (see Figure 4c).

Since we observed that only 11 out of 17 participants exhibited an increase in SO amplitude after the stimulation trigger, we performed a correlation analysis between ΔT1T0 percentage memory change and First‐type Stim Δpeaktopeak within the Stimulation group to determine whether participants with a greater ERP response to the stimulation showed a greater benefit in memory performance. The correlation analysis revealed a negative relationship between memory performance change and the SO response to stimulation, with participants showing a lower EEG response to the stimulation exhibiting higher memory performance (*r*_s15_ = −0.55, *p* = 0.021; see Figure S4).

## 4 Discussion

The present study aimed to assess the effects of CLAS in an ecological setting using a wearable EEG device. Specifically, we investigated whether one night of CLAS performed at participants' homes would benefit declarative memory performance and psychomotor vigilance. Additionally, we examined possible microstructural sleep changes resulting from the stimulation.

Our findings showed that CLAS modulated sleep microstructure, marked by a second SO after the first stimulation and a down‐state after the second stimulation. However, we found no effect on memory performance or psychomotor vigilance.

Regarding vigilance, no differences emerged between the Stimulation and Control groups. We did observe increased reaction times from T0 to T1 in the Control group, but this cannot be attributed to CLAS preventing vigilance decline, as PVT reaction times generally remain stable across sessions (Basner et al. 2018). Moreover, no correlation was found between sleepiness and vigilance, suggesting the cause of this increase is unclear and may reflect random variation. The lack of CLAS effects on vigilance aligns with prior laboratory‐based studies (Ngo et al. 2013; Schneider et al. 2020) and at‐home studies (Lustenberger et al. 2022), which also failed to find improvements, though some studies on sleep‐deprived participants did report benefits (Diep et al. 2021).

Similarly, no memory improvement was observed after stimulation or 2 days later. This contrasts with several laboratory‐based studies showing memory gains after CLAS during nocturnal (see Zhang and Gruber 2019) and diurnal sleep (Ong et al. 2016). However, our findings align with a portion of the existing literature, including both laboratory studies (Harrington et al. 2021; Henin et al. 2019; Ong et al. 2018) and at‐home studies (Diep et al. 2019) reporting no behavioural effects associated with CLAS. However, during training, the Stimulation group exhibited a steeper improvement compared to the Control group, although both reached similar performance levels at T0. This trend towards higher scores in the Stimulation group potentially masked an effect induced by the stimulation. Analyses on the differential scores (ΔT1T0) confirmed that no significant between‐group differences emerged, indicating comparable memory changes despite different learning trajectories. Although no cognitive benefits were seen, CLAS effectively modulated sleep microstructure, replicating findings from both laboratory settings during nocturnal sleep (Leminen et al. 2017; Ngo et al. 2013, 2015; Ong et al. 2016) and naps (Ong et al. 2018), as well as using wearable devices in home settings (Debellemaniere et al. 2018; Diep et al. 2021; Garcia‐Molina et al. 2018; Lustenberger et al. 2022).

We observed distinct effects depending on the stimulation type: both first and second stimulations triggered down‐states ~500 ms post‐stimulation, while the first also elicited a second up‐state ~1 s after stimulation. The second trigger also showed greater SO up‐state amplitude compared to the Sham condition.

Although CLAS enhanced SOs amplitude in some participants, stronger ERP responses were associated with poorer memory performance. This may suggest that a larger ERP response reflects increased arousal, which could interfere with memory consolidation (Bellesi et al. 2014). However, no increase in arousals was observed after stimulation compared to the previous night. These findings, however, should be interpreted with caution, given the small sample size. One possible explanation for the lack of memory benefit may lie in the nature of the task used. Our task (Salfi et al. 2024) has shown sleep‐related stability, with memory decaying little over time, even across 48 h. Given this, and the robust learning during training, CLAS may not have left any room to further enhance memory. The word‐pair task we used has previously been sensitive to CLAS effects (Leminen et al. 2017; Ngo et al. 2013, 2015; Ong et al. 2016; Papalambros et al. 2017), but ceiling effects may have limited detectable gains.

Of note, the evidence for CLAS's impact on memory performance is not entirely clear. Recent meta‐analyses (Harlow et al. 2023; Wunderlin et al. 2021) report overall small‐to‐moderate CLAS memory effects, though with high variability and declining effect sizes in recent years (Harlow et al. 2023). Some studies even report no or negative effects (e.g., Harrington et al. 2021; Schneider et al. 2020). For this reason, our small sample size, limited by equipment availability, may have further reduced power to detect small effects, increasing the risk of Type II error.

Another potential reason for the lack of cognitive benefit is that enhancing SOs alone may not suffice. Memory consolidation depends not only on SOs but also on their co‐occurrence with spindles and ripples (Staresina et al. 2023). Due to poor signal quality, we could not determine whether CLAS increased spindles or their coupling with SOs, leaving open the question of whether the full consolidation process was supported.

Our protocol had limitations. There was high variability in stimulation numbers across participants, driven by differences in total sleep time, N3 duration, and signal quality (see Table S4). We also lacked control over stimulation sound intensity (fixed at 45 or 55 dB), which may have been ineffective or disruptive depending on individual auditory thresholds or sleep depth (Bellesi et al. 2014; Esfahani et al. 2023). Moreover, the bone conduction stimulation method chosen by the Dreem Headband may present some limitations. Since the device is not firmly attached to the skin, any loss of contact between the stimulation system and the skin, such as movements during sleep or a change in position, could result in ineffective stimulation. Additionally, the intensity of the stimulation might differ from that typically achieved with loudspeakers. These issues may explain why about 26% of first stimulations (Stim 1 + Unique Stim) failed to evoke additional SOs (see Table S4), and in 6 of 17 participants, peak‐to‐peak ERP amplitude was no greater during Stim than Sham, indicating a weak or absent response in some cases (see Table S5). A further limitation of our study, although beyond our control, is the small sample size. This may have led to an underpowered study, preventing us from detecting a memory effect.

To conclude, the application of CLAS with an ambulatory EEG in an ecological setting successfully elicited sleep microstructure changes, evidenced by the generation of additional SOs in the EEG trace, without negatively affecting sleep architecture. However, this stimulation did not translate into measurable cognitive enhancements. The present study cannot draw firm conclusions about CLAS effects on memory, since our small sample size and the resulting limited statistical power may have prevented us from detecting a small‐to‐moderate effect size. Nevertheless, only one previous study has examined the effects of CLAS on memory in a home setting (Diep et al. 2019), reporting no memory benefits, in line with the present results. Therefore, further ecological studies are needed to investigate both the behavioural and physiological effects of CLAS.

## Author Contributions

**Angie Baldassarri:** data curation, formal analysis, investigation, software, visualisation, writing – original draft preparation. **Damiana Bergamo**, **Federico Salfi** and **Domenico Corigliano:** methodology, writing – review and editing. **Michele Ferrara:** writing – review and editing. **Aurora D'Atri:** writing – review and editing, funding acquisition. **Nicola Cellini:** conceptualisation, methodology, project administration, resources, supervision, validation, writing – review and editing, funding acquisition.

## Ethics Statement

All participants provided informed consent. The study protocol was approved by the local Ethics Committee (Comitato Etico della Ricerca Psicologica, area 17). Code: E1D8C482A4BD650BD2374B08DC4A7C67.

## Conflicts of Interest

The authors declare no conflicts of interest.

## Supporting information


**Table S1:** Statistical results of the model on the psychomotor vigilance task (PVT).
**Figure S1:** Predicted marginal effects of the psychomotor vigilance task (PVT) reaction times in the two groups across the two Testing phases. Bigger dots show the estimated mean reaction time. Smaller dots represent the observed data. Bars represent standard errors.
**Figure S2:** Mean probability of correct answers for word–pseudoword pairs in the two groups (Control, Stimulation) during the training phase and T0. The dots represent the mean probability across subjects. Bars represent standard errors.
**Table S2:** Statistical results of the model on the training trajectory.
**Table S3:** Statistical results of the model on testing sessions.
**Table S4:** Mean and standard deviation of the total number and percentage of stimulations, sham and unique triggers.
**Table S5:** Differences between up‐to‐down peak‐to‐peak amplitude (Δpeaktopeak) between First‐type Stim and First‐type Sham conditions for each participant.
**Figure S3:** Change in word–pseudoword pairs accuracy between T1 and T2 (ΔT2T1) in the two groups. Each dot represents a participant's observed data. The black dot represents the mean value. The box indicates the interquartile range (IQR), and the horizontal line is the median. Bars outside the box represent 1.5 × IQR.
**Figure S4:** Scatterplot showing the relationship between Δpeaktopeak in the First‐type Stim trigger and ΔT1T0 percentage accuracy in the Stimulation group. The shaded area represents the standard error. Each dot represents a participant; blue dots indicate participants with ΔT1T0 greater than 100, meaning they showed improvement at T1.
**Figure S5:** Polar plot showing the mean stimulation phase. The violet line indicates the mean phase across participants. Each blue line represents a participant's mean phase. Lines length reflects the vector strength. 0° corresponds to the SO up‐state.

## Data Availability

The data that support the findings of this study are openly available in OSF (Open Science Framework) at https://osf.io/as7wg/, reference number 10.17605/OSF.IO/AS7WG.
