# Supplementary material for: The Effect of Closed‐Loop Auditory Stimulation on Memory Consolidation and Sleep Physiology in an Ecological Setting
Source: J Sleep Res. 2025 Nov 12;35(3):e70247. doi: 10.1111/jsr.70247 (PMC13193352; doi:10.1111/jsr.70247)
Supplement: Supplementary file 1 — Table S1: Statistical results of the model on the psychomotor vigilance task (PVT). Figure S1: Predicted marginal effects of the psychomotor vigilance task (PVT) reaction times in the two groups across the two Testing phases. Bigger dots show the estimated mean reaction time. Smaller dots represent the observed data. Bars represent standard errors. Figure S2: Mean probability of correct answers for word–pseudoword pairs in the two groups (Control, Stimulation) during the training phase and T0. The dots represent the mean probability across subjects. Bars represent standard errors. Table S2: Statistical results of the model on the training trajectory. Table S3: Statistical results of the model on testing sessions. Table S4: Mean and standard deviation of the total number and percentage of stimulations, sham and unique triggers. Table S5: Differences between up‐to‐down peak‐to‐peak amplitude (Δpeaktopeak) between First‐type Stim and First‐type Sham conditions for each participant. Figure S3: Change in word–pseudoword pairs accuracy between T1 and T2 (ΔT2T1) in the two groups. Each dot represents a participant's observed data. The black dot represents the mean value. The box indicates the interquartile range (IQR), and the horizontal line is the median. Bars outside the box represent 1.5 × IQR. Figure S4: Scatterplot showing the relationship between Δpeaktopeak in the First‐type Stim trigger and ΔT1T0 percentage accuracy in the Stimulation group. The shaded area represents the standard error. Each dot represents a participant; blue dots indicate participants with ΔT1T0 greater than 100, meaning they showed improvement at T1. Figure S5: Polar plot showing the mean stimulation phase. The violet line indicates the mean phase across participants. Each blue line represents a participant's mean phase. Lines length reflects the vector strength. 0° corresponds to the SO up‐state. [file JSR-35-e70247-s001.docx]

Supplementary Material

The Effect of Closed-Loop Auditory Stimulation on Memory Consolidation and Sleep Physiology in an Ecological Setting

Angie Baldassarri^1^, Damiana Bergamo^1^, Federico Salfi^2^, Domenico Corigliano^2^, Michele Ferrara^2^, Aurora D’Atri^2^, Nicola Cellini^1,3,4^*

^1^Department of General Psychology, University of Padua, Padua, Italy

^2^Department of Biotechnological and Applied Clinical Sciences, University of L’Aquila, L’Aquila, Italy

^3^Padova Neuroscience Center, University of Padua, Padua, Italy

^4^Human Inspired Technologies Research Center, University of Padua, Padua, Italy

**Table S1.** Statistical results of the model on the psychomotor vigilance task (PVT)

|  | **PVT** | | | | | |  |  |
| --- | --- | --- | --- | --- | --- | --- | --- | --- |
| *Coefficient* | *Estimate* | | *SE* | *CI (95%)* | *z* | *p* |  |  |
| Intercept | 317.08 | | 7.90 | 301.96 – 332.96 | 231.11 | **<0.001** |  |  |
| GroupStimulation | 1.01 | | 0.04 | 0.94 – 1.08 | 0.26 | 0.797 |  |  |
| SessionT1 | 1.02 | | 0.01 | 1.01 – 1.04 | 3.11 | **0.002** |  |  |
| GroupStim:SessionT1 | 0.98 | | 0.01 | 0.96 – 1.00 | -1.79 | 0.073 |  |  |
| **Random Effects** | | | | | | | | |
| σ^2^ | | 0.02 | | | | | | |
| τ_00_ _ID_ | | 0.00 | | | | | | |
| ICC | | 0.06 | | | | | | |
| N _ID_ | | 34 | | | | | | |
| Observations | | 3226 | | | | | | |
| Marginal R^2^ / Conditional R^2^ | | 0.003 / 0.061 | | | | | | |

Notes. Estimate: exponentiated coefficient; SE: standard error; CI: confidence intervals set at 95%; z: z-score, p: p-value. P-values < .05 are represented in bold.

**
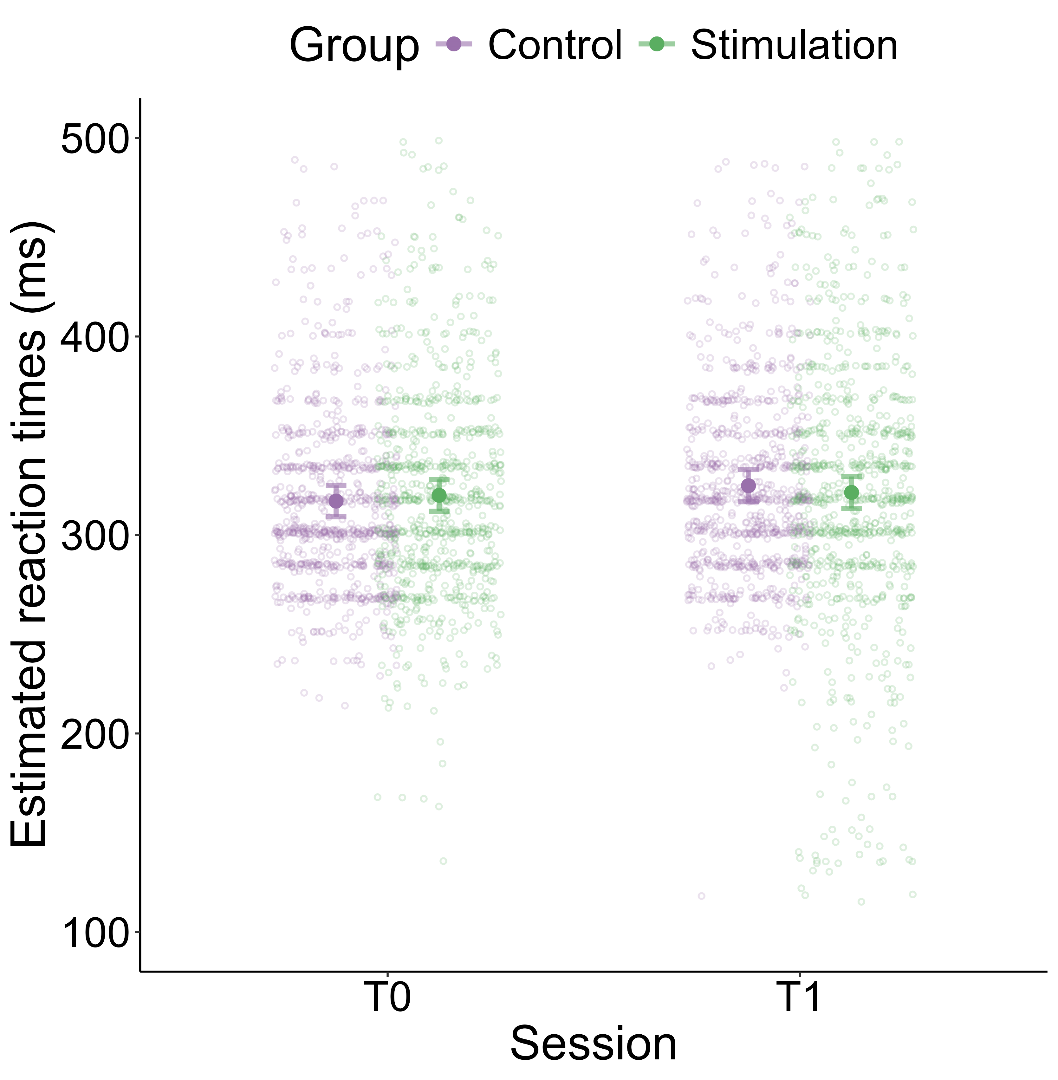
Fig. S1.** Predicted marginal effects of the psychomotor vigilance task (PVT) reaction times in the two groups across the two Testing phases. Bigger dots show the estimated mean reaction time. Smaller dots represent the observed data. Bars represent standard errors

**Training trajectory**

The model conducted on word-pseudoword pairs during the Training phase and T0 revealed improved performance as the number of sessions progressed, with post-hoc analysis showing a higher probability of correct answers in all sessions (Training 1, Training 2, Training 3, T0) (all p’s < .001). No difference between groups was observed at Training 1. The interaction Group × Session showed a greater improvement in the Stimulation group compared to the Control group from Training 1 to Training 2, from Training 1 to Training 3, and from Training 1 to T0. However, from post-hoc analysis, there was no difference between groups within any session (all p’s > .582). Although the Stimulation group showed a greater improvement over time, this difference did not translate into a significant difference with the Control group within sessions, indicating that both groups improved significantly throughout the Training phases, achieving a similar accuracy at T0 (Fig. S2). Full model results can be found in Table S2.

**
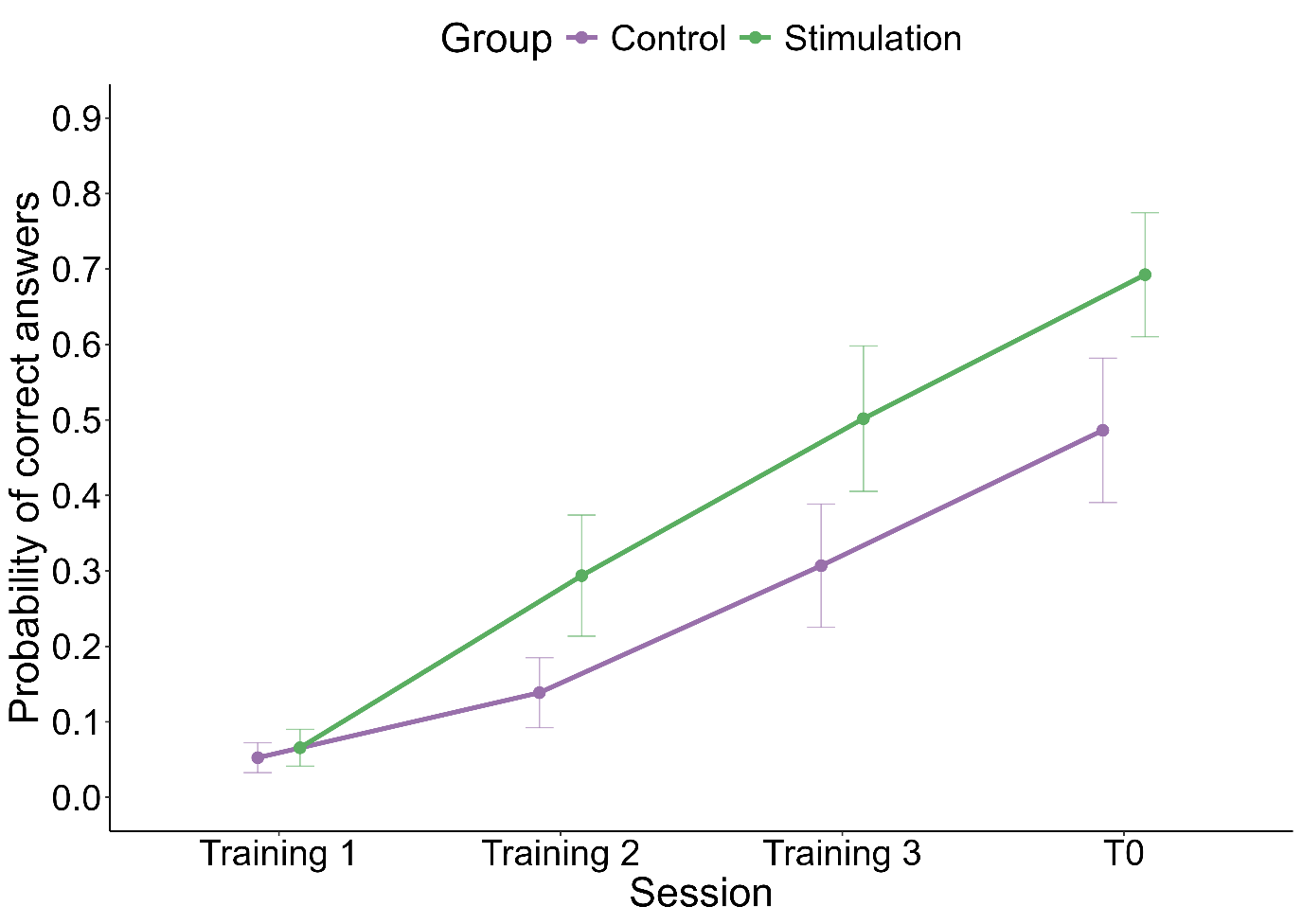
Fig. S2.** Mean probability of correct answers for word-pseudoword pairs in the two groups (Control, Stimulation) during the Training phase and T0. The dots represent the mean probability across subjects. Bars represent standard errors

**Table S2.** Statistical results of the model on the training trajectory

|  | **Training trajectory** | | | | | |  | |  | |
| --- | --- | --- | --- | --- | --- | --- | --- | --- | --- | --- |
| *Coefficient* | *Estimate* | *SE* | *CI (95%)* | *z* | | *p* |  | |  | |
| Intercept | 0.06 | 0.02 | 0.03 – 0.12 | -7.24 | | **<0.001** |  | |  | |
| GroupStim | 1.27 | 0.69 | 0.44 – 3.68 | 0.44 | | 0.662 |  | |  | |
| SessionTraining2 | 2.90 | 0.51 | 2.06 – 4.10 | 6.05 | | **<0.001** |  | |  | |
| SessionTraining3 | 7.99 | 1.37 | 5.71 – 11.17 | 12.15 | | **<0.001** |  | |  | |
| SessionT0 | 17.08 | 2.95 | 12.17 – 23.97 | 16.41 | | **<0.001** |  | |  | |
| GroupStim:SessionTraining2 | 2.04 | 0.50 | 1.26 – 3.29 | 2.90 | | **0.004** |  | |  | |
| GroupStim:SessionTraining3 | 1.79 | 0.43 | 1.11 – 2.88 | 2.41 | | **0.016** |  | |  | |
| GroupStim:SessionT0 | 1.88 | 0.46 | 1.16 – 3.04 | 2.55 | | **0.011** |  | |  | |
| **Random Effects** | | | | |  | | |  | |  |
| σ^2^ | 3.29 | | | |  | | |  | |  |
| τ_00_ _Word_ | 0.80 | | | |  | | |  | |  |
| τ_00_ _ID_ | 2.18 | | | |  | | |  | |  |
| ICC | 0.47 | | | |  | | |  | |  |
| N _ID_ | 34 | | | |  | | |  | |  |
| N _Word_ | 80 | | | |  | | |  | |  |
| Observations | 5440 | | | |  | | |  | |  |
| Marginal R^2^ / Conditional R^2^ | 0.196 / 0.578 | | | |  | | |  | |  |

Notes. Estimate: odds-ratio; SE: standard error; CI: confidence intervals set at 95%; z: z-score, p: p-value. P-values < .05 are represented in bold

**Table S3.** Statistical results of the model on testing sessions

|  |  | | | | **Testing sessions** | | | |
| --- | --- | --- | --- | --- | --- | --- | --- | --- |
| *Coefficient* | *Estimate* | | *SE* | *CI (95%)* | *z* |  | | *p* |
| Intercept | 0.96 | | 0.43 | 0.40 – 2.32 | -0.09 |  | | 0.926 |
| GroupStim | 2.61 | | 1.61 | 0.78 – 8.75 | 1.56 |  | | 0.119 |
| SessionT1 | 0.96 | | 0.13 | 0.74 – 1.25 | -0.27 |  | | 0.787 |
| SessionT2 | 1.02 | | 0.14 | 0.78 – 1.32 | 0.14 |  | | 0.893 |
| GroupStim:SessionT1 | 0.97 | | 0.19 | 0.65 – 1.43 | -0.16 |  | | 0.871 |
| GroupStim:SessionT2 | 0.87 | | 0.17 | 0.59 – 1.28 | -0.72 |  | | 0.474 |
| **Random Effects** | | | | | | |  | |
| σ^2^ | | 3.29 | | | | |  | |
| τ_00_ _Word_ | | 1.24 | | | | |  | |
| τ_00_ _ID_ | | 3.03 | | | | |  | |
| ICC | | 0.56 | | | | |  | |
| N _ID_ | | 34 | | | | |  | |
| N _Word_ | | 80 | | | | |  | |
| Observations | | 4080 | | | | |  | |
| Marginal R^2^ / Conditional R^2^ | | 0.026 / 0.576 | | | | |  | |

Notes. Estimate: odds-ratio; SE: standard error; CI: confidence intervals set at 95%; z: z-score, p: p-value. P-values < .05 are represented in bold

**Table S4.** Mean and standard deviation of the total number and percentage of stimulations, sham, and unique triggers

|  | M±SD (N) | Range | M±SD (%) |
| --- | --- | --- | --- |
| STIMULATION | 332.6±137.7 | 79-532 | 77.7±0.9 |
| shaM | 95.0±38.4 | 20-154 | 22.2±0.9 |
| UNIQUE STIMULATION | 47.5±20.4 | 11-84 | 26.5±9.7 |

Notes. The total number of stimulation triggers includes the combined counts of Stim 1, Stim 2, and Unique Stim. The total number of sham triggers includes the combined counts of Sham 1, Sham 2, and Unique Sham. Stimulations and Sham percentages represent the proportion of Stim and Sham triggers relative to the total number of triggers. The percentage of Unique Stim represents their occurrence relative to the total number of First-type triggers

**Table S5.** Differences between up-to-down peak-to-peak amplitude (Δpeaktopeak) between First-type Stim and First-type Sham conditions for each participant

| **1** | 234.12 |
| --- | --- |
| **2** | 59.58 |
| **3** | 208.12 |
| **4** | 171.77 |
| **5** | 147.04 |
| **6** | 80.67 |
| **7** | 124.75 |
| **8** | 91.27 |
| **9** | 46.17 |
| **10** | 128.18 |
| **11** | 147.75 |
| **12** | 141.93 |
| **13** | 193.13 |
| **14** | 93.61 |
| **15** | 121.73 |
| **16** | 133.08 |
| **17** | 97.55 |

Notes. Values over 100% indicate a higher peak-to-peak amplitude during the stimulation condition compared to the sham condition and are considered evidence of a successful manipulation. Values under 100% indicate a higher amplitude during the sham condition

**Difference in memory performance in the long term**


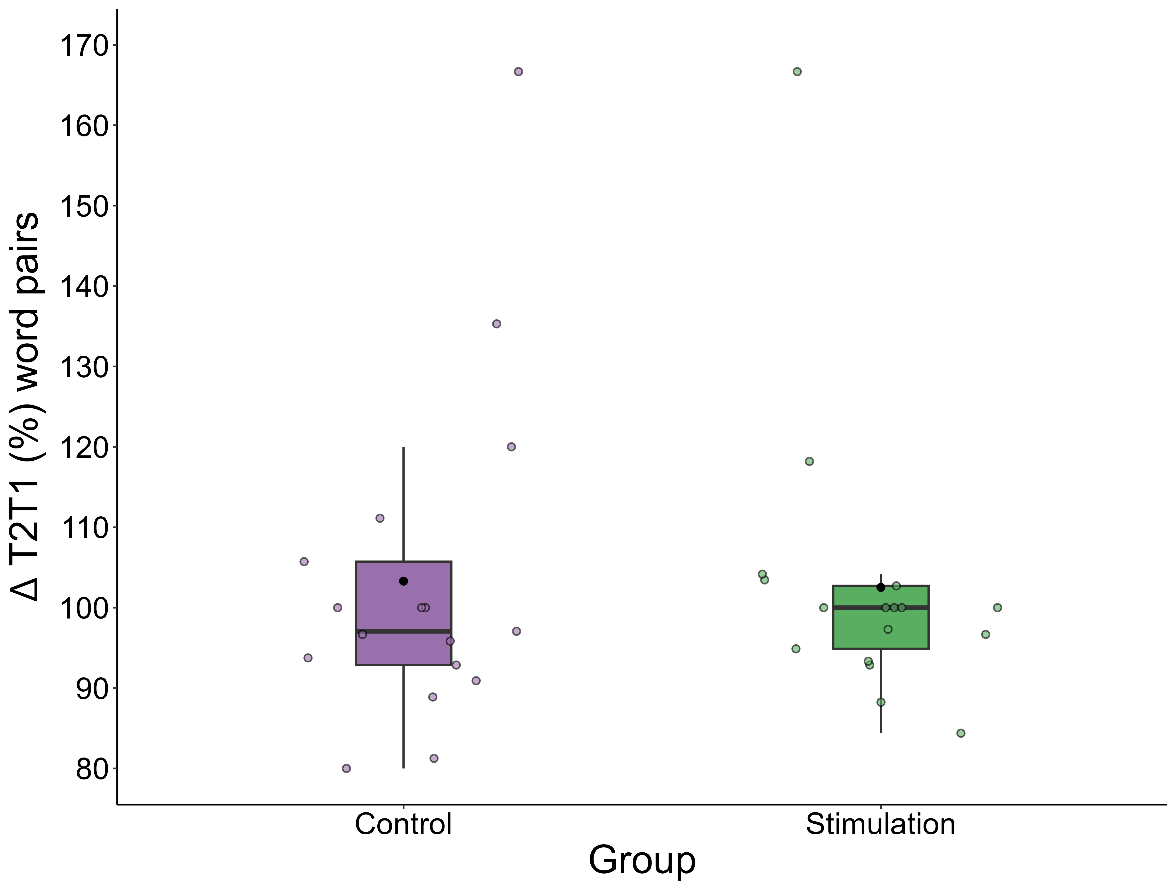
The Mann-Whitney test comparing ΔT2T1 accuracy for word-pseudoword pairs in the Stimulation and Control group did not show any significant difference (U = 155, p = .728, r = 0.06; Fig. S3).

**Fig. S3.** Change in word-pseudoword pairs accuracy between T1 and T2 (ΔT2T1) in the two groups. Each dot represents a participant’s observed data. The black dot represents the mean value. The box indicates the interquartile range (IQR), and the horizontal line is the median. Bars outside the box represent 1.5*IQR


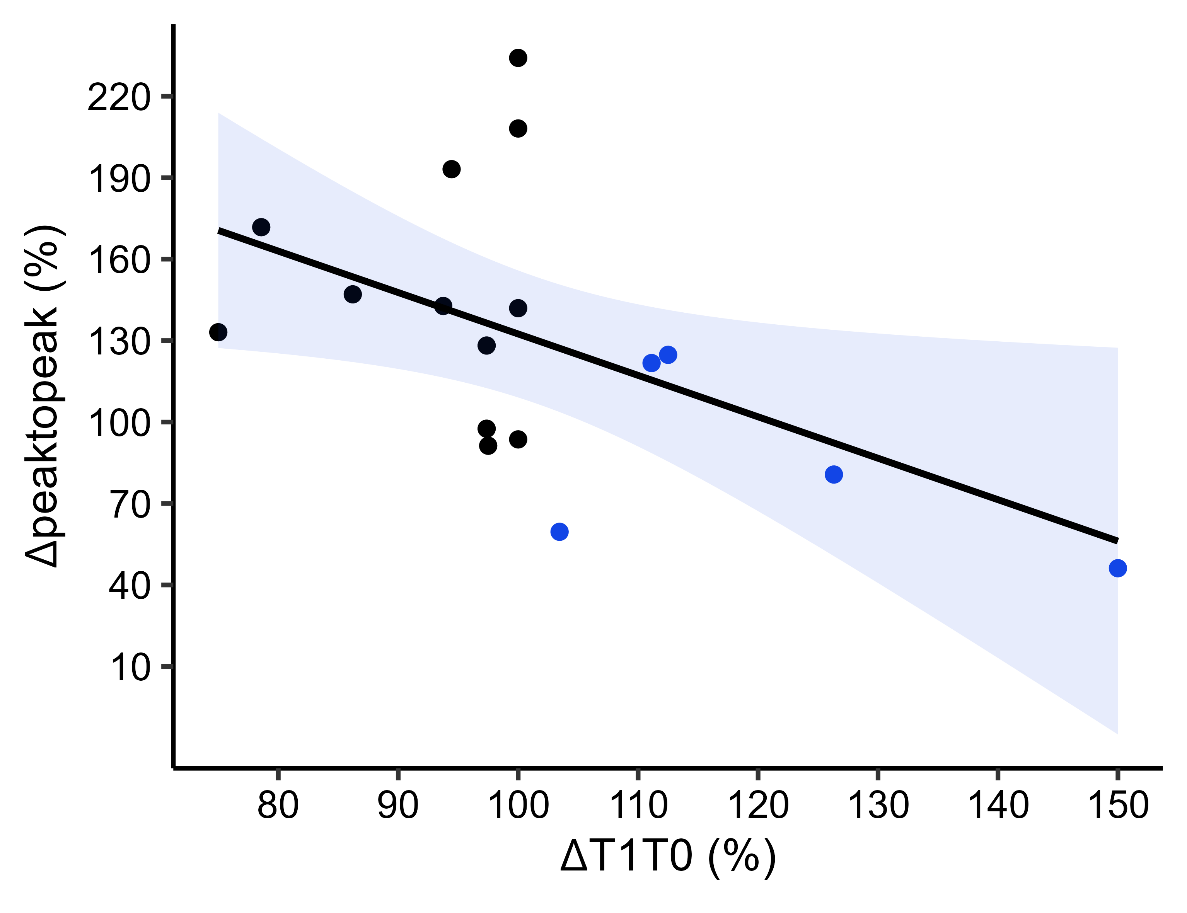


**Fig. S4.** Scatterplot showing the relationship between Δpeaktopeak in the First-type Stim trigger and ΔT1T0 percentage accuracy in the Stimulation group. The shaded area represents the standard error. Each dot represents a participant; blue dots indicate participants with ΔT1T0 greater than 100, meaning they showed improvement at T1

**Perceived sleepiness and cognitive performance**

The correlation between mean PVT performance and KSS outcomes did not show a relationship between the two measurements, either at T0 (r_τ_ = 0.02, p = .843) or at T1 (r_τ_ = 0.05, p = .713). Additionally, the KSS outcome did not show any relationship with memory performance at T0 (r_τ_ = -0.15, p = .234).

**Arousal in the Stimulation group**

The comparison of micro-arousals between the adaptation and experimental nights in the Stimulation group showed no significant difference (W = 82, p = .817, r = 0.06).

**Stimulation phase**


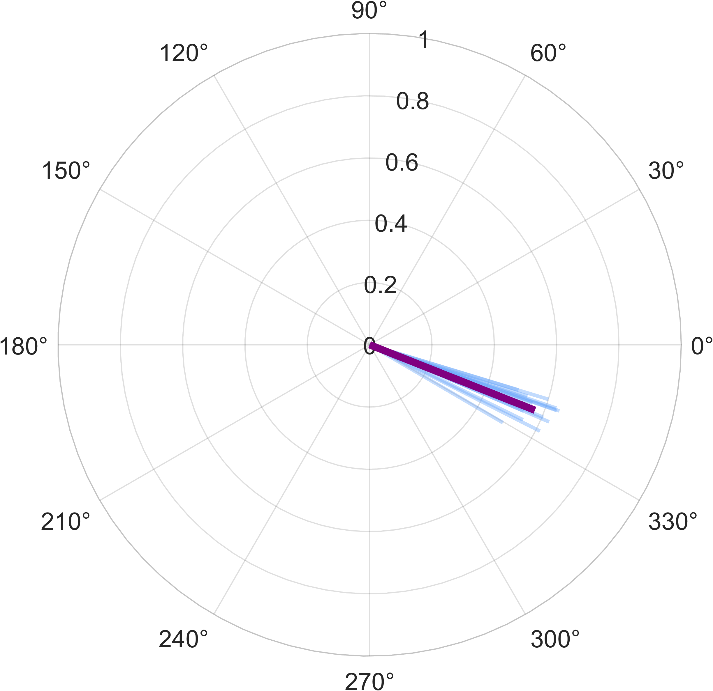
To verify the phase of the slow oscillation targeted by the stimulation, we conducted a phase analysis of the Stimulation triggers in the Stimulation group (N = 17). The F7–O1 signal was low-pass filtered at 1.25 Hz, as in Staresina and colleagues (2023), using a zero-phase FIR filter to avoid phase distortions. No high-pass filter was applied since the Dreem headband signal is already filtered at 0.4 Hz. Then, we applied the Hilbert transform within each 12-s epoch, and the instantaneous phase at each stimulation trigger was extracted using the *angle* function in MATLAB. For each participant, the mean phase was computed with *circ_mean* and the resultant vector length r with *circ_r* (Berens, 2009) to quantify the consistency of phases around the mean direction. For each participant, we performed the Rayleigh test using the *circ_rtest* function to test for non-uniformity of phases within participants. The test indicated that phases were not uniformly distributed (range of z values = 115.6 - 214.8, all p’s < .001). At the group level, the mean phase was 338.4°±1.4° (0° is the slow oscillation up-state). The vector length was 0.6, indicating good consistency across participants (see Fig. S5). A V test for non-uniformity was computed to assess if the mean phase among participants had a specific direction. The test against 0° (up-state) confirmed significant phase-locking around the up-state (v = 15.8, p < .001).

**Fig. S5.** Polar plot showing the mean stimulation phase. The violet line indicates the mean phase across participants. Each blue line represents a participant’s mean phase. Lines length reflects the vector strength. 0° corresponds to the SO up-state.

**References**

Berens, P. (2009). **CircStat**: A *MATLAB* Toolbox for Circular Statistics. *Journal of Statistical Software*, *31*(10). https://doi.org/10.18637/jss.v031.i10

Staresina, B. P., Niediek, J., Borger, V., Surges, R., & Mormann, F. (2023). How coupled slow oscillations, spindles and ripples coordinate neuronal processing and communication during human sleep. *Nature Neuroscience*, *26*(8), 1429–1437. https://doi.org/10.1038/s41593-023-01381-w
